# CT Imaging Patterns and Clinical Outcomes of Traumatic Brain Injury: Distribution Across Injury Mechanisms

**DOI:** 10.3390/jcm15187132

**Published:** 2026-09-14

**Authors:** Iulia T. Lupascu, Costin A. Minoiu, Bogdan V. Popa, Corina S. Homentcovschi, Lucian M. Florescu, Sorin Hostiuc

**Affiliations:** 1 Department of Legal Medicine and Bioethics, “Carol Davila” University of Medicine and Pharmacy Bucharest, 050474 Bucharest, Romania; iulia-tatiana.lupascu@drd.umfcd.ro; 2 Clinical Emergency Hospital Bucharest, 014461 Bucharest, Romania; 3 Department of Radiology, “Carol Davila” University of Medicine and Pharmacy Bucharest, 050474 Bucharest, Romania; 4 Department of Medical Semiology, “Carol Davila” University of Medicine and Pharmacy Bucharest, 050474 Bucharest, Romania; 5 Clinical Hospital Coltea, 030167 Bucharest, Romania; 6 Department of Radiology, University of Medicine and Pharmacy of Craiova, 200349 Craiova, Romania; 7 Clinical Emergency Hospital Craiova, 200642 Craiova, Romania; 8 The National Institute of Forensic Medicine “Mina Minovici”, 077160 Bucharest, Romania; 9 Department of Legal Medicine and Bioethics, Faculty of Dental Medicine, Carol Davila University of Medicine and Pharmacy, 020021 Bucharest, Romania

**Keywords:** traumatic brain injury, polytrauma, neuroimaging, cranial CT, intracranial hemorrhage

## Abstract

**Background:** Our goal was to evaluate the radiological patterns and clinical outcomes of brain injuries related to trauma, offering insight into the distribution of brain injuries, facial fractures and outcomes across road traffic accidents (RTAs), falls and other causes. **Methods:** Cerebral CT scans of 536 cases obtained between January 2019 and January 2024 were retrospectively reviewed. The collected data included demographics, etiology, transportation groups (driver, car occupant, pedestrian, motorcyclist, bicyclist), GCS (Glasgow Coma Scale), clinical outcome (ICU (intensive care unit) admission, neurosurgical intervention, mortality) and CT imaging features. Images were analyzed independently by two physicians. The findings reflect a selected population of polytrauma patients who underwent cerebral CT at a single emergency hospital. **Results:** Brain injury was identified in 39% of all subjects, the most frequent type of lesion being intraparenchymal hematoma (24%), followed by subdural hematoma (23%). Brain injury was numerically more frequent among patients with falls (45%) than among those involved in RTAs (37%), although this difference did not reach statistical significance in either unadjusted or age-, sex-, and mechanism-adjusted analysis (*p* > 0.05). CT patterns differed across transportation categories, with bicyclists showing associations with intraparenchymal hemorrhage and diffuse brain swelling and pedestrians with intraventricular hemorrhage (*p* < 0.05). However, given the small bicyclist subgroup (n = 6), these findings should be considered exploratory. Car occupants showed lower unadjusted odds of brain injury compared with drivers (OR: 0.50; 95% CI: 0.30–0.80), but this association was attenuated and no longer statistically significant after adjusting for age and sex (adjusted OR: 0.55; 95% CI: 0.27–1.15; *p* = 0.112). In multivariable analysis of in-hospital mortality, only age and severe GCS category remained independently associated with death; neither injury mechanism nor transportation category showed an independent association once these factors were accounted for. **Conclusions:** Brain injury was more frequently observed among patients with falls than among those involved in RTAs. Nevertheless, road traffic accidents accounted for the majority of polytrauma admissions. These findings highlight the importance of both road traffic safety measures and fall-prevention strategies, particularly among older adults.

## 1. Introduction

Traumatic brain injury (TBI) represents a major health concern worldwide, often accompanied by long-term consequences which can affect the quality of life [1]. Although TBI represents the outcome of multiple causal relationships, the two leading causes of TBI are traffic-related injuries and low- and high-level falls [2]. Falls are responsible for 49% of TBI-related visits to the emergency department in children under 17 and adults over 65 [3]. Collisions involving motor vehicles cause more than 1 million deaths and 50 million injuries reported each year. Work-related injuries, assaults and sports are also listed as common causes of TBI [3].

Some reports state that in the early 2000s, traffic regulations such as seatbelt laws, helmet wearing, airbags and lateral protection bars as well as the advancement of vehicle safety together managed to reduce the number of deaths from traffic accidents and also the rate and complexity of facial fractures [4]. However, more recent studies have mentioned an increase in road traffic injuries and fatalities caused by high speed, intoxication or distracted driving due to increased smartphone usage [5,6]. Depending on their severity, TBIs are classified into three categories—mild or concussion, moderate and severe—with 80% of cases being categorized as mild. However, mild TBI can also lead to debilitating symptoms [1]. The Glasgow Coma Scale is often used to describe those with TBI, with a mild score being 14–15, moderate 9–12 and severe 3–8 [7]. The standard initial evaluation of TBI includes non-contrast head computed tomography (CT) scanning as it is widely available and allows the rapid assessment of pathology that might require prompt surgical intervention [8]. Magnetic resonance imaging (MRI), however, has superior sensitivity for detecting diffuse axonal injuries and minor contusions. In cases of vascular abnormality, noninvasive angiography (CT angiography or MR angiography) may be required to describe vascular axes [9].

There are many studies evaluating the epidemiology and consequences of injuries caused by road traffic accidents (RTAs) [10,11,12]. However, comprehensive analysis is needed to focus on specific types of transportation, such as car drivers, occupants, pedestrians, motorcyclists and bicyclists, and their relationship with injuries. As each group shows unique injury patterns and clinical outcomes, targeted prevention strategies can be developed and medical resources can be optimally allocated within trauma care systems [13].

In this study, we aimed to evaluate the relationships between particular causes of traumatic brain injury and clinical and radiological patterns of brain injury or facial fractures. The research offers understanding of the distribution of brain injuries, facial fractures, clinical outcomes across transportation groups (bicyclist, motorcyclist, motor vehicle driver, occupant and pedestrian), falls and other causes. Facial fractures were assessed as a secondary outcome because they frequently co-occur with traumatic brain injury and share overlapping mechanisms, offering complementary insight into the overall craniofacial trauma burden across transportation groups.

## 2. Materials and Methods

This study was performed with the approval of the Ethics Institutional Review Board, under no. 9525/17 October 2023. Data collection was conducted in February 2024, following IRB approval, and included patients presenting to the emergency department between January 2019 and January 2024.

We conducted a retrospective study on polytrauma patients who presented at the emergency department of Clinical Emergency Hospital Bucharest from January 2019 to January 2024. The General Electric CT Optima 660 (GE Healthcare, Milwaukee, WI, USA) with 128 slices was used to perform cerebral CT scans, which included parenchymal and bone windows, with a slice thickness of 1.25 mm or 2.5 mm. For the majority of patients, reformatted images in the sagittal and coronal planes were obtained. The CT images were independently reviewed by two physicians: a board-certified radiologist and a senior radiology resident. Both readers were blinded to the clinical data but not to the injury mechanism at the time of image review. Discrepancies were resolved by consensus. The inter-rater agreement was not formally quantified, which is acknowledged as a limitation.

The eligibility criteria for inclusion were age equal to or over 17 and polytrauma patients, who were defined as patients with at least two injuries who had at least a non-contrast cerebral CT performed in our unit. The exclusion criteria consisted of patients who did not have a cerebral CT performed and patients with CT performed in another medical unit. Therefore, the study population represents polytrauma patients who underwent cerebral CT at our institution, rather than a comprehensive sample of all trauma, traumatic brain injury or road traffic accident cases; patients who died before CT could be performed or who were managed at other facilities are not represented. The polytrauma definition did not incorporate a validated severity score such as the Injury Severity Score (ISS) or Abbreviated Injury Scale (AIS).

The complete medical record of each patient fulfilling the inclusion criteria was reviewed and the following data were collected using Microsoft Excel software (last version no. 2408): age, sex, mechanism of trauma (road traffic accident, fall and different causes (in which we included work-related accidents, assaults and others); others were considered less frequent causes such as forest accidents, tractor or household accidents, or “not informed”; falls included accidental or suicidal falls from a height and falls from the same height. Other data reviewed included the GCS, evolution of the patient (admitted to the medical ward or intensive care unit, neurosurgical decompression, death) and imaging features of the injury on CT.

Age was analyzed both as a continuous variable and after categorization into five predefined groups: <30, 30–45, 46–60, 61–75 and >75 years. The general mechanism of injury was categorized as road traffic accident (RTA), fall or other causes. For patients involved in RTAs for whom this information was available, the transportation category was classified as driver, car occupant, pedestrian, motorcyclist or bicyclist.

The maxillofacial fractures were divided based on location: frontal sinus fracture, nasal fracture, maxillary fracture, zygomatic fracture and mandibular fracture.

The brain injuries evaluated were subdural hematoma, epidural hematoma, subarachnoid hemorrhage (SAH), intraventricular hemorrhage, intraparenchymal hemorrhage, pneumocephalus, diffuse brain swelling and brain herniation. Brain herniation has been considered subfalcine herniation, defined as a middle line shift of more than 5 mm.

Each brain injury was evaluated qualitatively as a dichotomous variable: yes/no. No radiomics features were extracted from CT images.

Because individual patients could present with multiple brain lesions and/or facial fractures, percentages describing specific injury and fracture subtypes were calculated using the total number of recorded lesions or fractures as the denominator.

Patient-level prevalence (e.g., the proportion of patients with any brain injury) is distinguished throughout from lesion-level distribution (e.g., the relative frequency of each injury subtype among all recorded lesions), and each table and figure caption specifies which denominator applies.

The transportation groups included drivers, car occupants, pedestrians, motorcyclists and bicyclists. Based on the GCS, patients were divided into three groups: group I: 3–8 scores, accounting for severe head injury; group II: 9–12 scores, representing moderate head injury; and group III: 13–15 scores, mild head injury.

The SPSS (Statistical Package for Social Sciences) software program, version 26 was used for statistical analyses. According to the type of data, the following tests were used: descriptive tests, chi-square tests and regression analysis. Categorical variables are summarized as counts and percentages, and continuous variables as means ± standard deviations. Univariable associations between categorical variables were assessed using the chi-square test (or Fisher’s exact test where the expected cell counts were below 5), with odds ratios and 95% confidence intervals calculated from 2 × 2 contingency tables. To account for potential confounding, multivariable logistic regression was additionally performed for two primary outcomes: (1) the presence of any brain injury, and (2) in-hospital mortality. The covariates included age, sex and injury mechanism (road traffic accident, fall or different causes; road traffic accident as reference) in both models, with the Glasgow Coma Scale category (mild, moderate, severe; mild as reference) additionally included in the mortality model. A secondary multivariable analysis restricted to road traffic accident patients with known transportation category examined transportation category (driver as reference) as a predictor of brain injury and mortality, adjusted for age and sex (and GCS category for mortality). The bicyclist subgroup was excluded from the transportation category mortality model due to zero observed deaths. No correction for multiple comparisons was applied, given the exploratory nature of the study; reported associations, particularly those from univariable analyses, should be interpreted as hypothesis-generating. Missing data were handled by variable-specific case deletion. The missing-data patterns for each variable are summarized in Supplementary Table S1. Patients with and without available data did not differ significantly by age or sex for etiology, GCS or ICU admission (all *p* > 0.05). Patients with missing mortality data differed significantly by sex (*p* = 0.025); this should be considered when interpreting mortality-related findings, though the absolute number of missing cases (n = 14) is small. A *p*-value of less than 0.05 was considered statistically significant.

## 3. Results

### 3.1. Study Population Characteristics

**Figure 1:**
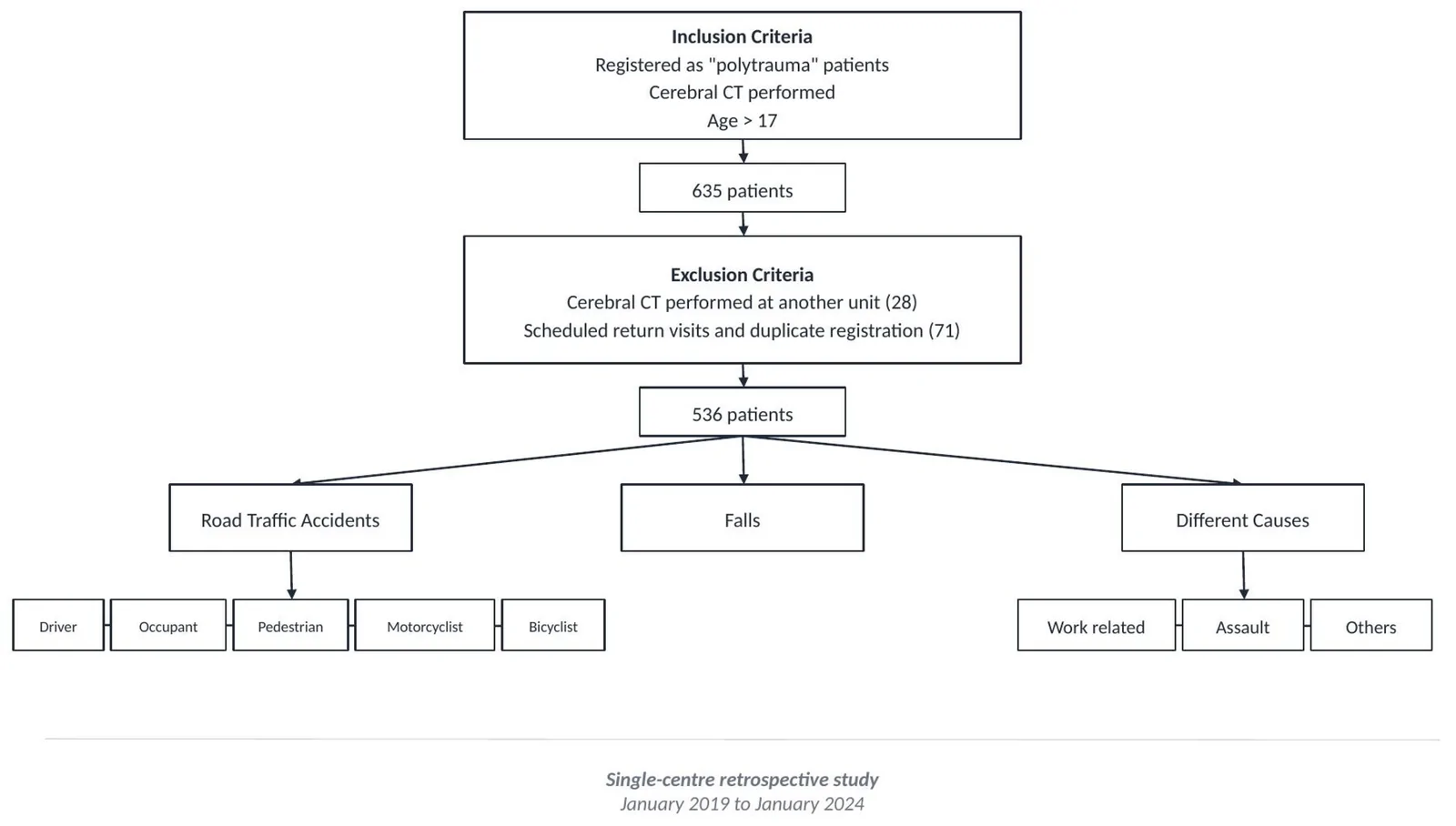
Flowchart of the inclusion process, showing the inclusion and exclusion criteria, the number of patients who were excluded in each step, the final number of patients and the main causes of polytrauma.

We included 536 subjects (Figure 1), of which 69% (n = 370) were males and 31% (n = 166) were females, with ages ranging from 17 to 92 and a mean age of 46 ± 17.5. Most subjects were part of the 46-to-60-year-old category (27%, n = 143), with males being mostly between 30 and 45 years (28%, n = 103) and females being mostly between 46 and 60 years (30%, n = 50) (Table 1). The mean age was 45.5 ± 17.2 years among males and 47.3 ± 18.1 years among females, with no statistically significant difference between the two groups (*p* = 0.285).

### 3.2. Etiology

Etiology information was available for 490 of the 536 patients, with 46 cases having missing data and being excluded from etiology-specific analyses, according to the predefined case deletion approach. Among patients with available etiology data, the main cause of polytrauma was RTAs, in 79% (n = 385), followed by falls, in 16% (n = 80), and different causes, in 5% (n = 25).

**Figure 2:**
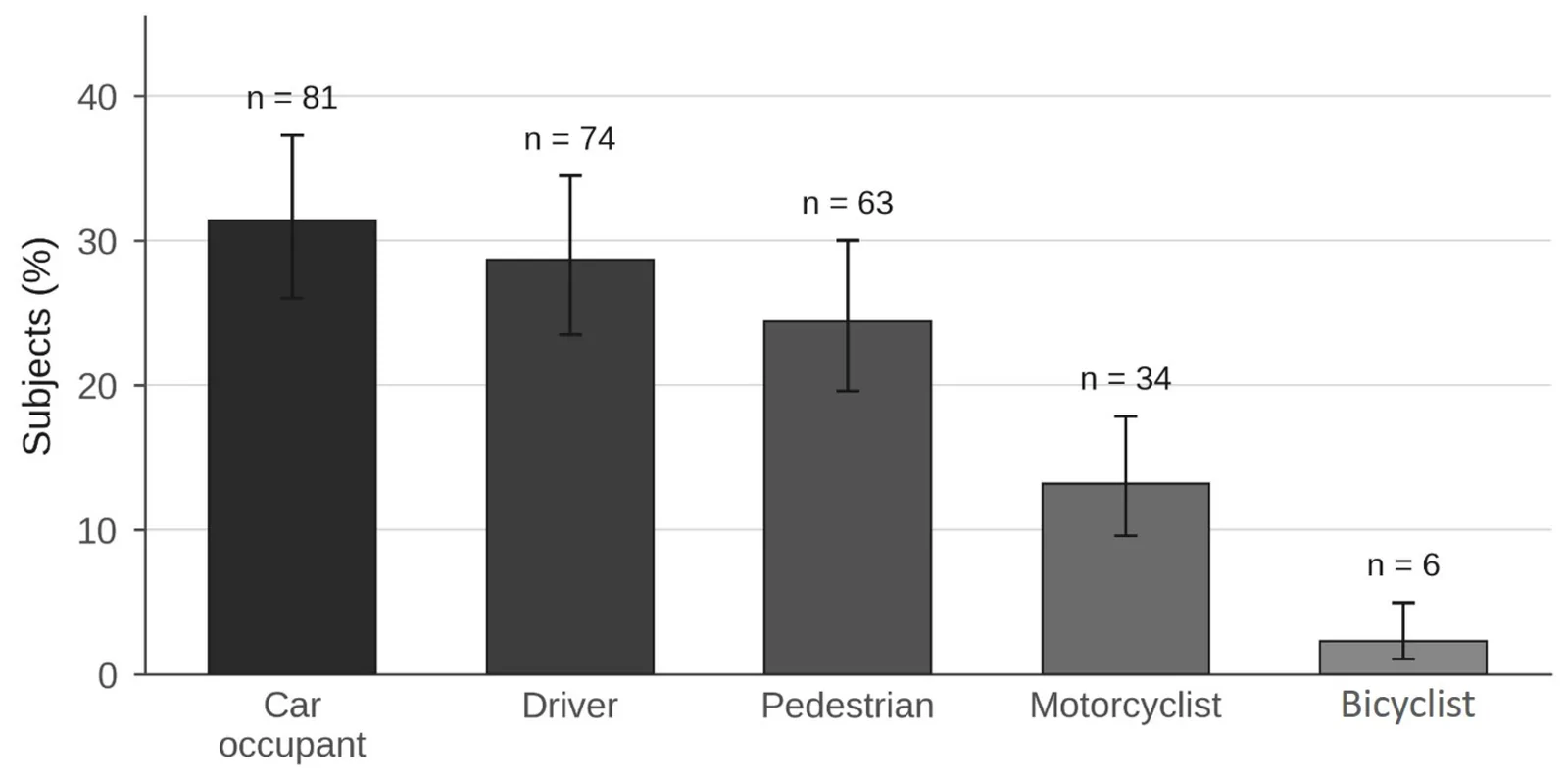
Distribution of transportation categories among patients involved in road traffic accidents. Percentages were calculated using patients with available transportation category data (n = 258) as the denominator.

Transportation category data was available for 258 of the 385 patients involved in road traffic accidents. Among these, the majority of subjects involved were car occupants at 31% (n = 81), followed by drivers at 29% (n = 74), pedestrians at 25% (n = 63), motorcyclists at 13% (n = 34) and bicyclists at 2% (n = 6) (Figure 2). Among the 25 patients classified as having different causes of injury, 13 were work-related accidents, 6 were assaults and 6 were classified as other causes.

Statistically significant associations were found between males and car drivers (*p* < 0.05; OR: 2.70; 95% CI: 1.40–5.20), males and motorcyclists (*p* < 0.05; OR: 3.70; 95% CI: 1.30–10.00), females and car occupants (*p* < 0.05; OR: 4.20; 95% CI: 2.60–7.00), and females and pedestrians (*p* < 0.05; OR: 1.90; 95% CI: 1.10–3.40). Conversely, statistically significant inverse associations were found between males and car occupants (*p* < 0.001; OR: 0.20; 95% CI: 0.10–0.30) and between females and car drivers (*p* < 0.05; OR: 0.30; 95% CI: 0.10–0.60).

There was a significant association between the subjects-under-30 category and RTAs (*p* < 0.05; OR: 1.70; 95% CI: 1.03–3.00), and 46–60 category and falls (*p* < 0.05; OR: 1.70; 95% CI: 1.05–2.90). Among the transportation groups, statistically significant associations were noticed: the under-30 category and motorcyclists (*p* < 0.001; OR: 4.40; 95% CI: 2.10–9.00), and the over-75 category and pedestrians (*p* < 0.05; OR: 3.20; 95% CI: 1.30–7.90). A statistically significant inverse association was observed between the 46–60-year age category and RTAs (*p* < 0.05; OR: 0.50; 95% CI: 0.30–0.80).

### 3.3. Hospital Admission

Among the 526 patients with available admission data, 75% (n = 393) of patients required ICU admission, while the remaining 25% (n = 133) of patients were admitted to a medical ward. Etiology information was available for 353 ICU-admitted patients, with road traffic accidents accounting for 80% (n = 281) of these cases, followed by falls in 16% (n = 56) and different causes in 4% (n = 16). Transportation category data were available for 188 of the 281 RTA patients admitted to the ICU. Among these, drivers accounted for 31% (n = 59) and occupants for 31% (n = 59), followed by motorcyclists at 12% (n = 22), pedestrians at 23% (n = 44) and bicyclists at 3% (n = 4), with no statistically significant association. Among patients with different causes of injury, 7 work-related accident cases, 3 assault cases and 6 cases classified as other causes required ICU admission. No significant association was found between hospital admission and mechanism of injury.

### 3.4. Associated Brain Injury

Brain injury was identified in 39% (n = 209) of all subjects and in 37% (n = 141) of subjects involved in RTAs, 45% (n = 36) of subjects involved in falls and 33% (n = 8) of subjects involved in different causes. The prevalence of brain injury within each transportation category was 41% (n = 30) among drivers, 26% (n = 21) across occupants, 29% (n = 10) across motorcyclists, 40% (n = 25) among pedestrians and 83% (n = 5) among bicyclists. Within the different causes category, brain injury was identified in 17% (n = 2) of work-related accidents, 33% (n = 2) of assaults and 67% (n = 4) of cases classified as other causes.

**Figure 3:**
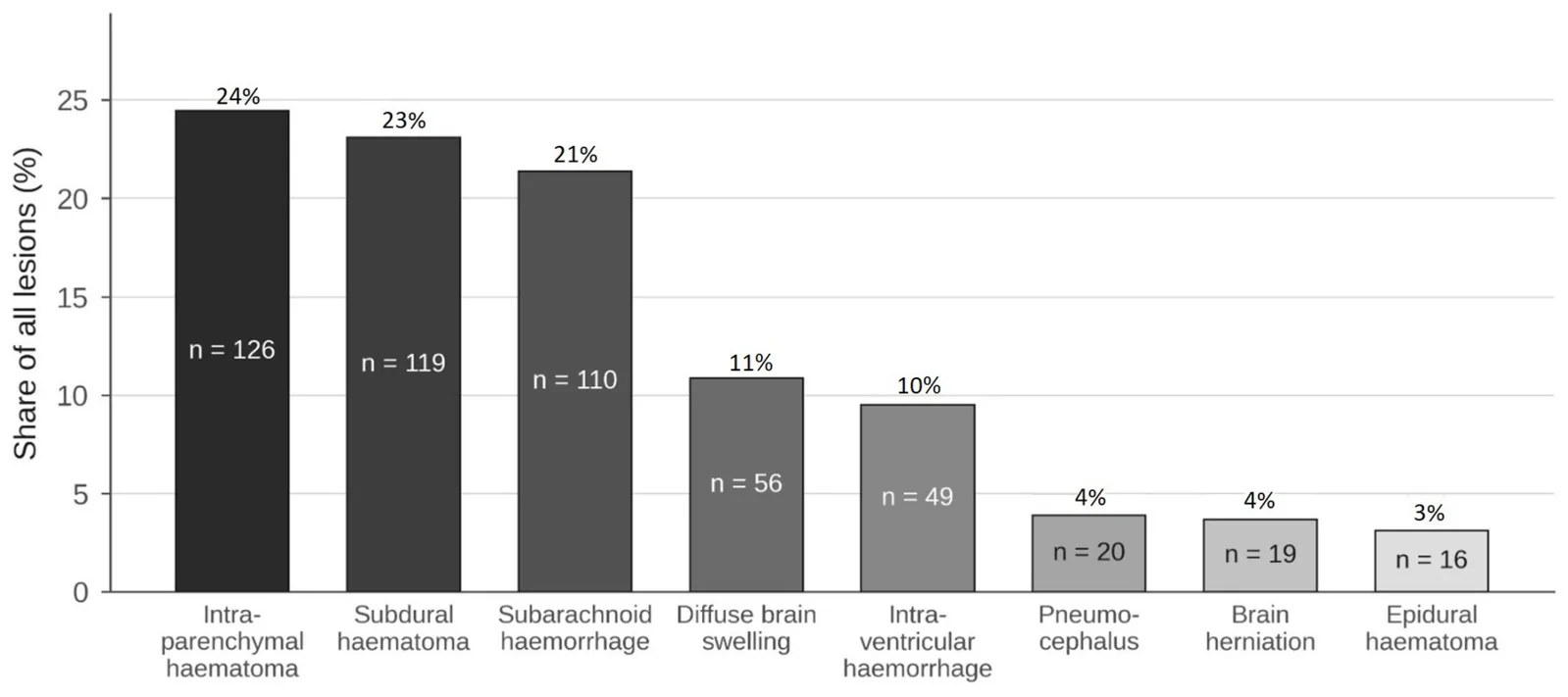
Distribution of brain injury types in polytrauma patients. Percentages were calculated using the total number of recorded brain injury findings (n = 515) as the denominator. Each injury type was recorded as present or absent for each patient; therefore, multiple lesions of the same type in a single patient were counted once, while patients with different types of brain injury contributed to more than one category.

The most frequent brain injuries identified in this study included intraparenchymal hematoma at 24% (n = 126), followed by subdural hematoma at 23% (n = 119), SAH at 21% (n = 110), diffuse brain swelling at 11% (n = 56), intraventricular hemorrhage at 10% (n = 49), pneumocephalus at 4% (n = 20), brain herniation at 4% (n = 19) and epidural hematoma at 3% (n = 16) (Figure 3).

The most frequently encountered brain injury among drivers was subdural hematoma at 23% (n = 17), followed by intraparenchymal hemorrhage at 19% (n = 14). Among car occupants, subdural hematoma was also the most encountered brain injury at 16% (n = 13), while intraparenchymal hemorrhage was the most common among motorcyclists at 21% (n = 7) and pedestrians at 22% (n = 14). A statistically significant association was observed between pedestrians and intraventricular hemorrhage (*p* < 0.05; OR: 2.70; 95% CI: 1.20–5.70), between bicyclists and intraparenchymal hematoma (*p* < 0.05; OR: 19.00; 95% CI: 1.90–143.00) and between bicyclists and diffuse brain swelling (*p* < 0.05; OR: 9.90; 95% CI: 1.90–50.00). A statistically significant inverse association was observed between car occupants and brain injury (*p* < 0.05; OR: 0.50; 95% CI: 0.30–0.80). However, in a multivariable model restricted to RTA patients with known transportation category and complete data for the variables included in the model (n = 256), after adjusting for age and sex, car occupants did not show significantly different odds of brain injury compared with drivers (adjusted OR: 0.55; 95% CI: 0.27–1.15; *p* = 0.112).

The most frequent type of brain injury associated with falls was intraparenchymal hemorrhage at 31% (n = 25), followed by SAH at 28% (n = 22). Among work-related accidents, intraparenchymal hemorrhage and diffuse brain swelling were the most frequent types of brain lesions, each accounting for 8% (n = 1) of cases. In assault-related injuries, subdural hematoma, SAH and intraparenchymal hemorrhage were equally represented, each occurring in 33% (n = 2) of cases. Other causes were most commonly associated with SAH and intraparenchymal hemorrhage at 67% (n = 4) and significantly associated with SAH (*p* < 0.05; OR: 5.40; 95% CI: 1.10–24.00). However, in multivariable analysis of the presence of any brain injury (n = 487), adjusting for age, sex and mechanism, no covariate reached statistical significance, including mechanism (falls vs. RTAs, adjusted OR: 1.40; 95% CI: 0.86–2.29; *p* = 0.179; different causes vs. RTAs, adjusted OR: 0.82; 95% CI: 0.34–1.97; *p* = 0.654), age (adjusted OR: 0.99 per year; 95% CI: 0.98–1.01; *p* = 0.336) or sex (adjusted OR: 1.23; 95% CI: 0.82–1.84; *p* = 0.310).

### 3.5. Associated Facial Fractures

Of all the subjects, 26% (n = 139) presented with facial fractures. The most frequently encountered facial fractures included the zygoma at 28% (n = 69), followed by the maxillary bone at 27% (n = 66), nasal bones at 23% (n = 58), frontal sinus at 13% (n = 32) and mandible at 9% (n = 22). Facial fractures were encountered in 23% (n = 90) of subjects involved in RTAs, 24% (n = 19) of subjects with falls and 24% (n = 6) of subjects with different causes.

Among the transportation groups, drivers most often had maxillary bone fracture at 31% (n = 11), followed by zygoma fracture at 28% (n = 10); car occupants had maxillary bone fracture and zygoma fracture equally frequently at 32% (n = 7); motorcyclists had maxillary bone fracture at 44% (n = 4); pedestrians had zygoma fracture most often at 35% (n = 7); and bicyclists had equally frequent fractures of the nasal bones, zygoma and frontal sinus at 33% (n = 1). The most frequently encountered facial fractures due to work injuries were nasal bones at 23% (n = 3), followed by the frontal sinus at 15% (n = 2). In assaults, the most common facial fracture was in the zygoma bone at 50% (n = 3). No facial fractures were encountered in the “others” group. There was no significant association between facial fractures and causes.

### 3.6. Clinical Outcome

GCS data were available for 390 patients. The majority of subjects presented with a mild GCS score (56%, n = 218), followed by severe (30%, n = 117) and moderate GCS scores (14%, n = 55). Neurosurgical decompression was performed in 4% (n = 20) of cases and midline shift was identified in 3.5% (n = 19) of subjects.

Overall, 15% (n = 81) of the subjects died in the hospital. Etiology data were available for 73 deceased patients. Of these, 79% (n = 58) were involved in road traffic accidents, 15% (n = 11) in falls and 6% (n = 4) in different causes of injury. Admission to the ICU was significantly associated with death in unadjusted analysis (*p* < 0.05; OR: 16.00; 95% CI: 4.00–69.00).

Regarding survival according to the mechanism of injury, 17% (n = 12) of drivers died in the hospital, as did 8% (n = 6) of occupants, 20% (n = 12) of pedestrians, 9% (n = 3) of motorcyclists, no bicyclists, 8% (n = 1) of those involved in work-related injuries, 17% (n = 1) of those involved in assaults and 33% (n = 2) of those with other causes. The mechanism was not independently associated with mortality once age, sex and GCS were accounted for (falls vs. RTAs, adjusted OR: 0.92; 95% CI: 0.38–2.20; *p* = 0.846; different causes vs. RTAs, adjusted OR: 0.92; 95% CI: 0.26–3.24; *p* = 0.897). Within the road traffic accident subgroup, no transportation category was independently associated with mortality after adjustment for age, sex and GCS category (all *p* > 0.25); the bicyclist subgroup (n = 6, zero deaths) was excluded from this model due to complete separation.

Seventeen percent (n = 29) of the females and 15% (n = 52) of the men died, without a statistically significant difference. There was a significant association between women over 75 years old and death (*p* < 0.05; OR: 10.00; 95% CI: 2.80–39.00) and between death and the age categories of 61–75 (*p* < 0.05; OR: 2.40; 95% CI: 1.40–4.10) and over 75 years old (*p* < 0.05; OR: 2.70; 95% CI: 1.20–6.30). In multivariable analysis of in-hospital mortality (n = 352), adjusting for age, sex, mechanism and GCS category, only age (adjusted OR: 1.03 per year; 95% CI: 1.01–1.05; *p* = 0.002) and severe GCS category (adjusted OR: 9.32; 95% CI: 4.69–18.54; *p* < 0.001, versus mild) remained significantly associated with death.

## 4. Discussion

In this study, we aimed to evaluate CT imaging patterns and clinical outcomes in polytrauma patients with traumatic brain injury, with a focus on differences in injury patterns across transportation groups (bicyclist, motorcyclist, motor vehicle driver, occupant and pedestrian), falls and different causes.

The initial imaging tool used for assessment is a CT scan of the brain without contrast, due to its high availability. Skull X-rays are usually recommended for evaluating stab wounds, gunshots or foreign bodies [3]. The role of CT in trauma is recognized in the postmortem context as well, as it can identify fracture sites and foreign bodies and can show the gas distribution, useful aspects in both RTAs and gunshot wounds [14,15]. As a limitation, CT gives a macroscopic view of the brain, without enabling us to diagnose changes like diffuse axonal injury. On the other hand, MRI is not usually used as an initial tool for evaluation, since it is less available compared to CT, requires a longer time for scanning and also requires safety screening for metallic foreign objects or medical devices that are not compatible. However, MRI is more sensitive for the detection of small non-hemorrhagic contusions and diffuse axonal injury and can also be used when there is a discrepancy between a patient’s neurological status and CT findings [9].

The mean age of our subjects was 46 ± 17.5 years, which is in agreement with newer studies that mention a mean age of trauma patients of 49 ± 43 years [16]. Some reports in the literature state that the age of the trauma population is increasing, as a reflection of the aging population, which is associated with frailty, multimorbidity and polypharmacy, all of these increasing the risk of falls and adverse outcomes [17]. The mean age of men was slightly lower than that of women, although the difference was not statistically significant. Similarly to other studies, men dominate the younger categories of age, while the majority of women in our study belong to the 46–60-years category [18].

We noticed an association between the under-30 category and RTAs and under-30 category and motorcyclists, which is in concordance with other studies from the literature [19]. A potential explanation for this is a tendency for high-risk conduct in this age category, inexperience, peer pressure and developmental changes with increased emotionality [20]. In contrast, RTAs were less frequently observed among patients aged 46–60 years. The majority of the subjects in this study were admitted to the ICU.

Brain injury was identified in 45% of the subjects involved in falls, and 37% of subjects involved in RTAs. New findings in the literature show that an epidemiologic shift has appeared in the last few years, with falls surpassing traffic accidents as the main cause of TBI [21]. Among transportation groups, brain injuries were encountered most frequently in bicyclists followed by car drivers. However, the bicyclist finding is based on a very small subgroup and should be considered hypothesis-generating rather than conclusive.

Nevertheless, other studies in the literature showed that non-helmeted cyclists have an increased risk of subdural hematoma and skull fracture [10], with a 54% increase in bicycle-related TBI between 1998 and 2012 [22]. This also underlines a limitation of our study, as it does not include information regarding personal protective equipment such as helmets. We noticed an association between bicyclists and intraparenchymal hematoma and bicyclists and diffuse brain swelling. However, the wide confidence interval highlights the uncertainty regarding the magnitude of the effect, most probably due to the small sample size in this group. In this regard, further research is needed with a larger number of patients.

We also found pedestrians to be significantly associated with intraventricular hemorrhage. Other studies found that craniocerebral injuries are predominant among motorcyclists and pedestrians, with car passengers being less susceptible to head trauma compared to the above-mentioned groups [23]. Similarly, in our study, being a car occupant was associated with lower odds of brain injury compared with the other transportation groups. However, when adjusted for age and sex, the association between being a car occupant and reduced odds of brain injury was attenuated and no longer statistically significant, suggesting that demographic differences between occupants and other road users, rather than seating position itself, may account for part of the unadjusted association. Nevertheless, Daskal Y. et al. showed that among car passengers, drivers have a lower risk of TBI compared to rear seat passengers and that there is no significant difference in regard to the risk of TBI between drivers and front seat passengers [24].

In our study, 26% of subjects presented facial fractures, which is similar to other data from the literature, where the incidence of facial injuries accompanying major trauma is up to 34% in Washington and between 15 and 24% in England [25]. The etiology of facial fractures varies among studies. Some mention motor vehicle accidents as the main reason for maxillofacial trauma in developing or under-developed countries and assaults in developed countries [26]. Our study showed RTAs to be the main etiology for facial fractures, followed by falls. This variation can perhaps be explained by the overpopulation of cities in under-developed countries, with high-risk driving behavior and poor traffic regulations [27].

Overall mortality was 15%, which is comparable with other similar studies [28], with the highest proportion of deaths occurring among patients involved in RTAs (79%), followed by falls (15%). We did not observe a significant difference in survival according to the mechanism of injury. The WHO 2023 mentions a category named vulnerable road users, which includes pedestrians and users of two wheels, a category which is involved in more than half of road traffic deaths [29]. Some studies have mentioned that pedestrians are the least likely to survive, followed by drivers, motorcyclists or cyclists and passengers/other injury types [30]. Our study supports this statement, with pedestrians and drivers being the least likely to survive. Other studies also found pedestrians and two-wheelers to represent the majority of fatalities among RTAs, with Kamabu K. et al. noticing that motorcyclists are 5.9-times more likely to die compared to pedestrians [31]. The mortality rates of trauma patients in our study increased with age, especially in the sixth and seventh decades of life. The strong association observed between ICU admission and in-hospital mortality in the unadjusted analysis should be interpreted cautiously, as ICU admission is closely related to injury severity and the association was not adjusted for potential confounders. No significant difference in survival was observed in regard to gender. On the contrary, Spoerri A. et al. showed there is a higher risk of death due to RTAs among men, and these results are supported by other studies in the literature [32,33,34,35]. However, we did notice a significantly higher death rate among the older women in the study, pertaining to the above-75-year-old category. Even after adjustment for age, sex and GCS category, no transportation category was independently associated with mortality; however, residual confounding by unmeasured factors such as extracranial injury burden or collision severity cannot be excluded.

Although the difference in brain injury prevalence between falls and RTAs did not reach statistical significance after adjustment, falls remained numerically associated with the highest prevalence of brain injury in our cohort, particularly relevant given the aging trauma population.

## 5. Limitations

The retrospective nature of this study represents a limitation. Associations between transportation category and CT findings or mortality were not adjusted for potential confounders such as collision speed, helmet or seatbelt use, alcohol involvement, anticoagulant therapy or extracranial injury burden, none of which were available in our dataset. The reported associations should therefore be interpreted as adjusted for the available covariates only, not as fully adjusted independent risk estimates.

The lack of MRI limits the spectrum of evaluated brain injuries, with no insight into the presence of diffuse axonal injuries. No thoraco-abdominal–pelvic injuries were included, which might have acted as confounders. ICU admission may partly reflect institutional triage practices rather than injury severity alone and mortality was assessed only during hospitalization, without post-discharge follow-up. Outcomes such as length of stay, ventilator use, discharge destination or 30-day mortality were not available and would strengthen future analyses.

Because inclusion required a cerebral CT performed at our institution, patients who died at the scene, were transferred without imaging or were evaluated elsewhere were not included, an aspect which limits the generalizability of our findings to broader trauma or TBI populations.

The extremely small bicyclist subgroup (n = 6, zero observed in-hospital deaths) precluded inclusion in the multivariable mortality model due to complete separation, and any associations involving this subgroup—including the previously reported associations with intraparenchymal hematoma and diffuse brain swelling—should be interpreted as hypothesis-generating only. Even though our results agree with other studies, further research should be conducted in multiple centers, with the inclusion of biomarkers and preinjury pathologies, in order to minimize the high variability that exists among literature studies about this complex topic.

## 6. Conclusions

Our study showed that the leading cause of polytrauma was RTAs, followed by falls. The main etiology for facial fractures was also represented by RTAs and most frequently included injuries to the zygoma and maxillary bone. Falls showed a numerically higher prevalence of brain injury than RTAs, although this difference did not reach statistical significance after adjustment for age, sex and mechanism. Associations between transportation category and brain injury or mortality did not persist after adjustment for age and sex, and should be interpreted as exploratory rather than definitive. Most subjects were admitted to the ICU. However, the strong unadjusted association between ICU admission and in-hospital death likely reflects injury severity rather than an independent effect. Patients younger than 30 years were significantly associated with RTAs. Since RTAs still represent the majority of polytrauma patients admitted to hospital, traffic regulations and prevention programs focused on seatbelts, helmets and the education of young adults remain a reasonable priority, although this cannot be directly demonstrated by the present dataset. Furthermore, since falls remain numerically associated with the highest prevalence of brain injury in our cohort, fall-prevention strategies for older adults, such as home safety assessment, balance training, medication review and management of osteoporosis, should be considered alongside road safety interventions.

## Figures and Tables

**Table 1:** Age distribution by gender.

Age Group (Years)	Males	Females	Total
<30	24% n = 90	23% n = 38	24% n = 128
30–45	28% n = 103	23% n = 38	26% n = 141
46–60	25% n = 93	30% n = 50	27% n = 143
61–75	18% n = 67	17% n = 29	18% n = 96
>75	5% n = 17	7% n = 11	5% n = 28

## Data Availability

The raw data supporting the conclusions of this article will be made available by the authors on request.

## Supplementary Materials

The following supporting information can be downloaded at: https://www.mdpi.com/article/10.3390/jcm15187132/s1. Table S1: Missing-data patterns by variable. For each variable, the number and percentage of subjects with missing data are reported, along with results of comparisons.

## Abbreviations

The following abbreviations are used in this manuscript:

TBI	Traumatic brain injury
RTA	Road traffic accidents
GCS	Glasgow Coma Scale
ICU	Intensive care unit
SAH	Subarachnoid hemorrhage
CT	Computed tomography
MRI	Magnetic resonance imaging
