# Supplementary material for: CT Imaging Patterns and Clinical Outcomes of Traumatic Brain Injury: Distribution Across Injury Mechanisms
Source: J Clin Med. 2026 Sep 14;15(18):7132. doi: 10.3390/jcm15187132 (PMC13607637; doi:10.3390/jcm15187132)
Supplement: Supplementary file 1 [file jcm-15-07132-s001.zip › jcm-4422985-supplementary.pdf]

Supplementary Table S1.

Table S1: Missing-data patterns by variable. For each variable, the number and percentage of subjects with missing data are reported, along with results of comparisons

Variable	No. of subjects	No. missing	% missing	p (age)	p (gender)
Mechanism (etiology)	491	45	8.4%	0.488	0.247
Transport category (only RTA, n=386)	258	128	33.2%	—	—
GCS category	390	146	27.2%	0.126	0.788
ICU / medical ward	526	10	1.9%	0.248	0.073
Neurosurgery	523	13	2.4%	—	—
Death (in-hospital)	522	14	2.6%	0.265	0.025 *
Brain injury (any)	530	6	1.1%	—	—
